# Gene-repaired iPS cells as novel approach for patient with *osteogenesis imperfecta*

**DOI:** 10.3389/fbioe.2023.1205122

**Published:** 2023-06-30

**Authors:** Agnieszka Fus-Kujawa, Barbara Mendrek, Karolina Bajdak-Rusinek, Natalia Diak, Karolina Strzelec, Ewa Gutmajster, Kamil Janelt, Agnieszka Kowalczuk, Anna Trybus, Patrycja Rozwadowska, Wojciech Wojakowski, Katarzyna Gawron, Aleksander L. Sieroń

**Affiliations:** ^1^ Department of Medical Genetics, Faculty of Medical Sciences in Katowice, Medical University of Silesia, Katowice, Poland; ^2^ Centre of Polymer and Carbon Materials, Polish Academy of Sciences, Zabrze, Poland; ^3^ Department of Molecular Biology, Faculty of Medical Sciences in Katowice, Medical University of Silesia, Katowice, Poland; ^4^ Biotechnology Centre, Silesian University of Technology, Gliwice, Poland; ^5^ Students Scientific Society, Faculty of Medical Sciences in Katowice, Medical University of Silesia, Katowice, Poland; ^6^ Division of Cardiology and Structural Heart Diseases, Medical University of Silesia, Katowice, Poland; ^7^ Formerly Department of Molecular Biology, Faculty of Medical Sciences in Katowice, Medical University of Silesia, Katowice, Poland

**Keywords:** induced pluripotent stem cells, osteogenesis imperfecta, star polymer, reprogramming, patient’s specific cell/gene therapy, homologous recombination, delivery systems

## Abstract

**Introduction:** The benefits of patient’s specific cell/gene therapy have been reported in relation to numerous genetic related disorders including osteogenesis imperfecta (OI). In osteogenesis imperfecta particularly also a drug therapy based on the administration of bisphosphonates partially helped to ease the symptoms.

**Methods:** In this controlled trial, fibroblasts derived from patient diagnosed with OI type II have been successfully reprogrammed into induced Pluripotent Stem cells (iPSCs) using Yamanaka factors. Those cells were subjected to repair mutations found in the *COL1A1* gene using homologous recombination (HR) approach facilitated with star polymer (STAR) as a carrier of the genetic material.

**Results:** Delivery of the correct linear DNA fragment to the osteogenesis imperfecta patient’s cells resulted in the repair of the DNA mutation with an 84% success rate. IPSCs showed 87% viability after STAR treatment and 82% with its polyplex.

**Discussion:** The use of novel polymer Poly[N,N-Dimethylaminoethyl Methacrylate-co-Hydroxyl-Bearing Oligo(Ethylene Glycol) Methacrylate] Arms (P(DMAEMA-co-OEGMA-OH) with star-like structure has been shown as an efficient tool for nucleic acids delivery into cells (Funded by National Science Centre, Contract No. UMO-2020/37/N/NZ2/01125).

## 1 Introduction

The breakthrough discovery of induced Pluripotent Stem cells (iPSCs) had meaningful influence on the use of these cells in cell/gene therapy. The application of iPSCs seems to be a valuable alternative for the treatment of many human diseases because, unlike Embryonic Stem Cells (ESCs), acquisition of these cells do not rise ethical controversies (Aach et al., 2017). The use of iPSCs as a source of patient-specific cells has been already proven for various degenerative diseases, mostly affecting single small or larger organs, such as ischemic heart failure, Parkinson’s, and Alzheimer’s disease, diabetes mellitus, and age-related macular degeneration (Malik and Rao, 2013; Doss and Sachinidis, 2019). Also, numerous clinical trials have been initiated to evaluate therapies for such disorders in terms of their efficacy and safety (Mandai et al., 2017; Cyranoski, 2018). Systemic disorders affecting numerous organs or entire organism, such as OI, sclerosis multiplex, and autoimmune aggression disorders still need to be addressed by cell/gene therapy approach.

Efficient and controlled delivery of nucleic acids using carriers with low toxicity is one of the most important challenges faced by gene therapy (Poddar et al., 2019). Interestingly, the possibility to repair mutations in genomic DNA (gDNA) of reprogrammed cells offers hope for patients suffering from incurable diseases that cannot be successfully treated with conventional methods. So far, many attempts have been made in iPSCs using retroviruses (Saito et al., 2018), adeno-associated virus 6 (AAV6) (Martin et al., 2019), baculoviruses (Zhu et al., 2013), and genetic conversion of SMN2 to SMN1 via CRISPR/Cpf1 and single-stranded oligodeoxynucleotides to correct genes, and increase the chance to obtain isogenic mutant lines, based on targeting therapeutic genes (Zhou et al., 2018; Paolini Sguazzi et al., 2021). In addition, the positive features of viral vectors include cell-specificity, which also determines their effectiveness and the lack of pathogenicity or infection of cells. The disadvantages include insertional mutagenesis or consequent tumorigenic potential, which may decrease the efficacy of viral vectors (Paolini Sguazzi et al., 2021).

The severe side effects caused by the immunogenic response to viral carriers require creation of safer, less pathogenic non-viral synthetic alternatives. Therefore, for the complexation of DNA or RNA cationic polymers may be an alternative, as they are able to electrostatically bind, condense and protect anionic nucleic acid chains within nanometer-sized particles, forming so called polyplexes.

Non-viral polymeric gene carriers have advantages over the viral vectors, as they offer structural and chemical versatility in terms of modifying physicochemical properties, low host immunogenicity, better storage stability and lower costs of manufacturing (Wong et al., 2007). Such polymers are able to interact with cell surface, trigger intracellular uptake and deliver the nucleic acid to the site of its action. The cytotoxicity of non-viral carriers, accumulation at the cell surface, the transport to the cell nucleus and DNA release still need improvement (Gao et al., 2007; Wong et al., 2007). Numerous polymers of different architectures (linear, branched, perfectly branched dendrimers), various chemical compositions and sizes, and their self-assemblies (micelles, aggregates) have been studied as potential vector candidates (Wong et al., 2007; Xu and Yang, 2011; Lächelt and Wagner, 2015). A number of study reports have shown that properties like molar mass, degree of branching, cationic charge density and polyplex surface charge, conformation in solution, and type of cationic functionality affect cytotoxicity and transfection efficacy (Park et al., 2006; Wong et al., 2007; Ganta et al., 2008).

Thus, concluding, efficient nucleus translocation or stable and specific gene expression support the use of cationic polymers. One of the most important disadvantages is their possible cellular cytotoxicity (Paolini Sguazzi et al., 2021). The use of polymers with star topology, containing a significant number of available reactive groups and lower cytotoxicity compared to their linear analogues, results in a satisfactory intensity of gene expression. Mainly cationic stars of polyethyleneimine (PEI) (Yang et al., 2007; Namgung et al., 2009), poly (N,N-dimethylaminoethyl methacrylate) (PDMAEMA) (Georgiou et al., 2004; Georgiou et al., 2005) and poly (N,N-dimethylaminopropylacrylamide) (Ishikawa et al., 2008) were used for polymer-nucleic acid complexes formation. Several groups have demonstrated that polyplexes formed by stars with nucleic acids exhibited higher transfection efficacy than their linear counterparts (Shim et al., 2008; Dai et al., 2010; Alhoranta et al., 2011; Zheng et al., 2013). Star polymers were used in prokaryote transformation (Souza et al., 2021) and transfection of the eukaryotic non-human like COS-1 (Dai et al., 2010; Zhou et al., 2007) and human cell lines such as HT-1080, HMEC, BdEC and others (Xu et al., 2009; Fus-Kujawa et al., 2020; Fus-Kujawa et al., 2022). Their use as gene delivery vectors has also included gene silencing (Mori et al., 2009; Boyer et al., 2013). Therefore, star polymers are a promising tool in gene therapy (Ren et al., 2016). These particles were also extensively tested as a platform for drug delivery and release, e.g. for paclitaxel, docetaxel or methotrexate (Wiltshire and Qiao, 2007; Ren et al., 2016; Yang et al., 2017). These polymers may also have an application in contrast-enhancing MRI medical imaging (Ren et al., 2016) and in tissue engineering (Fu et al., 2007; Bailey et al., 2012; Grover et al., 2014). They may also be used to develop antimicrobial (Liu et al., 2012) and implantable materials as another example of their biomedical application (Go et al., 2008; Groll et al., 2009).

The main goal of gene therapy is to achieve patient-specific cells with as high efficiency as it is possible. Lipofectamine is commonly used for co-transfection. It contains lipid groups that form so called liposomes in an aqueous environment. These groups have the ability to capture the charge of delivered nucleic acids. Lipid-mediated transfection is used for hard-to-transfect cells such as stem cells with high efficiency. It is possible to deliver DNA of all sizes. Additionally, it is non-toxic to cells which is important when considering the use of the biological material in *in vivo* therapies.

According to the researches, lipid systems for cells transfection are the perfect tool for siRNA and plasmid DNA delivery with 90% efficiency (Ejima et al., 2017). These systems have various compositions of lipids depending on the transfected cells. Importantly, it is an effective way of nucleic acid delivery that has no cytotoxic effect on dividing primary cells. It has been proven in *in vitro* and *in vivo* conditions. Formulations of lipopolyplexes have applications in gene therapy. The lipid component is important for the stability of the liposome and its electrostatic association with DNA formulating the lipopolyplex during transfection. Surprisingly, it does not involve in membrane interactions (Du et al., 2014). It has been also proven that some liposomes accelerate the phase transition when loaded with an other molecules/components such as drug (Le-Deygen et al., 2022).

The interactions of polyplexes (the complex of nucleic acids and polymer) with cell membranes are crucial during transfection. The molecular basis of this step is crucial for understanding the molecular mechanisms of polymer-mediated delivery of nucleic acid into the target cells. So far, the formation of polyplexes of DNA/siRNA with polymers has been assessed using the computer simulations. This has enabled the exploration of the structure and binding patterns of these components.

Importantly, free energy profiles for polyplexes bound to the membrane surface were estimated. Polycations are shown to have a protective effect on delivered DNA against dehydration. Higher polymer concentration is, more pronounced effect is observed (Gurtovenko, 2019).

To date, there is still no dataavailable showing the use of star polymers (STAR) to repair mutations in gDNA. The STAR polymer is an alternative to viral vectors that increase the immunogenicity of the obtained cells. In the present study, we have demonstrated the benefits of using a mixture of lipofectamine and STAR polymer for higher nucleic acids delivery efficiency.

The aim of the study reported in this work is to prove that cells from a patient with type II osteogenesis imperfecta (OI), a rare skeletal dysplasia (Deguchi et al., 2021), might be converted to pluripotent stem cells. The iPSCs can then be subjected to repair mutation in gDNA. The OI is an example of a systemic disorder, therefore it addresses the problem of curing systemic genetic related disorders with a wide spectrum of severity, but still associated with a specific manifestation, which is bone abnormalities. Moreover, the etiopathogenesis of OI is related to the presence of mutations in various gene, such as *CRTAP*, *LEPRE1* or *PPIB*. However, the most common gene mutations are *COL1A1* (17q21.31–22.05) and *COL1A2* (7q21.3–22.1), which account for more than 90% of cases (Galicka, 2012). Here we selected a lethal case due to abnormal collagen type I production in various tissues. Clinically it is manifested by brittle and deformed bones, a high incidence of fractures and growth deficiency (Forlino and Marini, 2016). In general, the severity of this disease ranges from mild to severe, with the form seen in the presented patient being perinatally fatal (Götherström et al., 2014). Mortality measured as survival time ranges from 1 year after birth for type II OI, several years with severe skeletal deformities for type III OI, to non-lethal with mild or asymptomatic skeletal abnormalities (Marini et al., 2007). The patient, whose cells were used in this work was diagnosed with OI type caused by two mutations found in the *COL1A1* gene that cause structural and quantitative deficiency of collagen type I (Marini et al., 2007). Although gene and cell therapies have been applied to prevent the expression of mutant alleles their efficacy was not satisfying and the results were not permanent (Wang et al., 1996). The pitfalls also concerned stem cell transplantation, which replaced osteoblasts producing defective collagen proteins with normal cells, but the replacement efficacy was less than 1.5% (Niyibizi and Li, 2009). The iPSCs can differentiate widely by expressing pluripotency genes. Therefore, they can differentiate into a wide range of cell types. On the other hand, non-viral transfection with star polymers can be used as a potential therapeutic solution for OI patients mainly due to their chemical structure, as well as molecular weight in relation to their effect on cytotoxicity (Bekhite and Schulze, 2021).Thus, in this work, we assessed the possibility of repairing two mutations in the *COL1A1* gene in patient-derived cells after their reprogramming to iPSCs. We have used star polymers to evaluate their use as non-viral carriers of genetic material in order to achive high efficacy of DNA mutation repair. Although this is preliminaty work on the use of non-viral DNA delivery vectors for gene repair and characterization of iPSCs as vectors for the correction of genetic disorders, it opens a new direction of research into the possibilities of cell/gene therapy in patients with OI and disorders alike (Otsu, 2015).

## 2 Materials and methods

### 2.1 Cell lines/Patients and sample collection

Human skin fibroblasts were obtained from 3-day-old newborn diagnosed with OI, type II (OMIM number: #166200). The procedure was in accordance with the protocol described elsewhere (Witecka et al., 2008; Gawron et al., 2017; Gawron et al., 2018). Mutation located in exon 44 (c.3155-3163del; g.18047 del gtgcccctg; p.Gly1052-1054 del; written as D4-Triple helix) of *COL1A1* was detected in the patient’s genomic DNA and deposited with reference number AN_002603 (https://www.le.ac.uk/ge/collagen/). Resequencing of gDNA in these cells revealed an additional mutation, causing a substitution C>T, (g:18062). The patient parents read and signed a written informed consent form prior to using the patient’s cells in the study. The study was carried out in accordance with the Declaration of Helsinki and was approved by the Bioethics Committee of the Jagiellonian University, Medical College in Krakow, Poland (KBET/108/B/2007). Human dermal fibroblasts (PDF) used as control cells were purchased from the American Type Culture Collection (ATCC, Manassas, VI, United States).

### 2.2 Use of Poly [N,N-Dimethylaminoethyl methacrylate-co-hydroxyl-bearing Oligo (Ethylene glycol) methacrylate] arms (P(DMAEMA-co-OEGMA-OH)

Star copolymer with poly(arylene oxindole) core and 28 poly[N,N′-dimethylaminoethyl methacrylate-co-hydroxyl-bearing oligo(ethylene glycol) methacrylate] arms (STAR) was obtained and characterized as previously described (Fus-Kujawa et al., 2020). In experiments a star copolymer with a molar mass of 100 000 Da (M_n_) and dispersity index (M_w_/M_n_) equal to 2.2 was used. The content of OEGMA-OH in the star (calculated from ^1^H NMR spectrum) was equal to 10%mol.

Cytotoxicity assay was performed in the range of star polymers’ concentrations: 0 (control) 5, 10, 20, 30, 40, 50, 60, 70, 80, 90, and 100 μg/mL and N/P ratios: 0, 1, 2, 3, 4, 6, 8, 16, 32, and 64). N/P ratio means the ratio of the number of amino groups in the polymer structure to the number of phosphate groups of nucleic acid. Cell viability was assessed by the percentage of viable polymer-treated cells compared to untreated control cells.

### 2.3 Generation of human iPSCs and functionality

At this stage of research the generation of iPSCs was conducted with a commonly used method utilizing Sendai virus expressing OCT4, SOX2, KLF4, and c-MYC (Yamanaka factors; OSKM) using CytoTune™-iPS 2.0 Sendai Reprogramming Kit (Thermo Fisher Scientific Inc.) according to the manufacturer’s instruction. On day 28 post-infection, the colonies were picked and transferred to a new vitronectin-coated plate for further culture.

### 2.4 Karyotyping

Karyotype has been prepared for human iPSC at passage 20. Cells at 30% of confluence were treated with colcemid (Sigma Aldrich, Germany) for 10 min. The metaphase chromosomes were analyzed after the G-banding stain. At least 20 metaphase spreads were analyzed for individual iPSC (Song et al., 2021).

### 2.5 Flow cytometry

Cells were fixed with 4% paraformaldehyde (Sigma aldrich, Germany) and incubated for 30 min at RT. Then, the cells were washed for 5 min with DPBS. For intracellular markers, cells were permeabilized with 0,2% Tween 20 (Thermofisher Scientific, United States) for 20 min at RT. After each step, the cells were washed twice with PBS for 5 min at RT. Subsequently, the cells were incubated with 2% FBS for 1 h at RT. Then, the proper primary antibody was added and incubated overnight at 4°C. After incubation time, the cells were washed twice with PBS and secondary antibody was added for 1 h (RT, in the dark).

### 2.6 Three germ layers differentiation

Important property of stem cells is their ability to differentiate to cells specific for the three germ layers. Therefore, the 3-germ layer immunocytochemistry kit (Thermo Fisher Scientific Inc.) was used. The procedure applied herein enables the detection of relevant germ layers markers, such as beta-III tubulin (TUJ1) for ectoderm, alpha-fetoprotein (AFP) for endoderm, and Smooth Muscle Actin (SMA) for mesoderm. The procedure has been performed according to the manufacturer’s instructions. Pictures were taken under inverted light microscope with fluorescence (OLYMPUS, Japan).

### 2.7 iPSCs-derived Mesenchymal stem cells (MSCs)

IPSCs-derived MSCs were obtained using STEMdiff Mesenchymal Progenitor Kit (Stemcell Technologies, Germany) according to the manufacturer’s instructions.

### 2.8 Bacterial strains

The strain of *Escherichia coli*, TOP10 (Invitrogen, CA) was used for amplification and DNA cloning of all DNA constructs.

### 2.9 Preparation of the correct linear DNA fragment

The genomic DNA (gDNA) encoding the collagen gene were isolated from human dermal fibroblasts. The DNA was purified with the use of genomic DNA purification BloodMini Kit (A&A Biotechnology, Poland) according to the manufacturer’s protocol. The purified DNA was used as a template to obtain DNA fragments by PCR. The DNA fragment was cut using *NotI* and *EcoRI* (Thermo Fisher Scientific Inc.).

### 2.10 Preparation of plasmid DNA

The plasmid used for cloning was pcDNA3.1 (-) vector carrying both ampicillin and neomycin resistance genes (Thermo Fisher Scientific Inc.). DNA was purified using the Plasmid DNA Maxi Kit (Omega Bio-Tek, Inc., United States). pDNA was cut using *NotI* and *EcoRI* (Thermo Fisher Scientific Inc.).

### 2.11 DNA mutation repair

To evaluate the percentage of transfected iPSC, 1 × 10^6^ cells were seeded onto vitronectin-coated 100 mm Petri dish. On another day, cells were transfected with polyplexes mixed with Lipofectamine™ Stem Transfection Reagent (Thermo Fisher Scientific Inc.) according to the manufacturer’s instructions. For this purpose, polyplexes consisting of STAR at a ratio of N/P = 64, correct DNA fragment (cut with *NotI* and *EcoRI*) and pDNA (pcDNA3.1 (-)) (cut with *NotI* and *EcoRI*) (the ratio of free ends of pDNA to the correct DNA fragment was 1:200) diluted in Opti-MEM I Medium were incubated for 30 min at room temperature (RT). Subsequently, diluted Lipofectamine™ Stem Transfection Reagent was mixed with polyplexes and incubated for 10 min at RT at the ratio 1:1. Then, the mixture has been added to the cells. After 24 h, the medium was replaced, and the cell culture was continued for another 24 h. Then, antibiotic G418 (G418 disulfate salt, Sigma aldrich, Germany) was added to a final concentration of 350 μg/mL. The iPSC with G418 antibiotic at a concentration of 350 μg/mL was selected for 48 h. After this time, the concentration of antibiotic G418 was reduced to 150 μg/mL and selection continued for another 14 days. The medium was changed every 3 days. Following this, the transfected iPSCs were detached with 50 mM EDTA (Thermo Fisher Scientific Inc.) solution and seeded onto 48-well plates at a density of one viable cell per well for clonal proliferation. When 80% confluence of iPSC in a well was achieved, the cells were seeded onto two wells to obtain cells for gDNA isolation.

For transfection efficiency assay, the cells were seeded at a density of 1 × 10^6^ cells per 100 mm vitronectin-coated Petri dishes. Three variants of transfection were analyzed: a) STAR only, b) STAR + lipofectamine, c) lipofectamine only. Above-mentioned conditions were applied. The number of living cells was calculated with Alamar blue test. The non-transfected iPSCs constitute the positive control.

### 2.12 Isolation of DNA and DNA sequencing

The gDNA was isolated from transfected iPSCs clones, resistant to G418, using QIAamp DNA Blood Mini Kit (QIAGEN, Germany) to analyze the sequencing products with the use of Genetic Analyzer ABI3130XL (Applied Biosystems, WA, United States). Primers used for DNA sequencing are listed in Supplementary Table S1.

## 3 Results

### 3.1 iPSCs obtained from human dermal fibroblasts with a mutation in COL1A1 reveal pluripotency state

**FIGURE 1:**
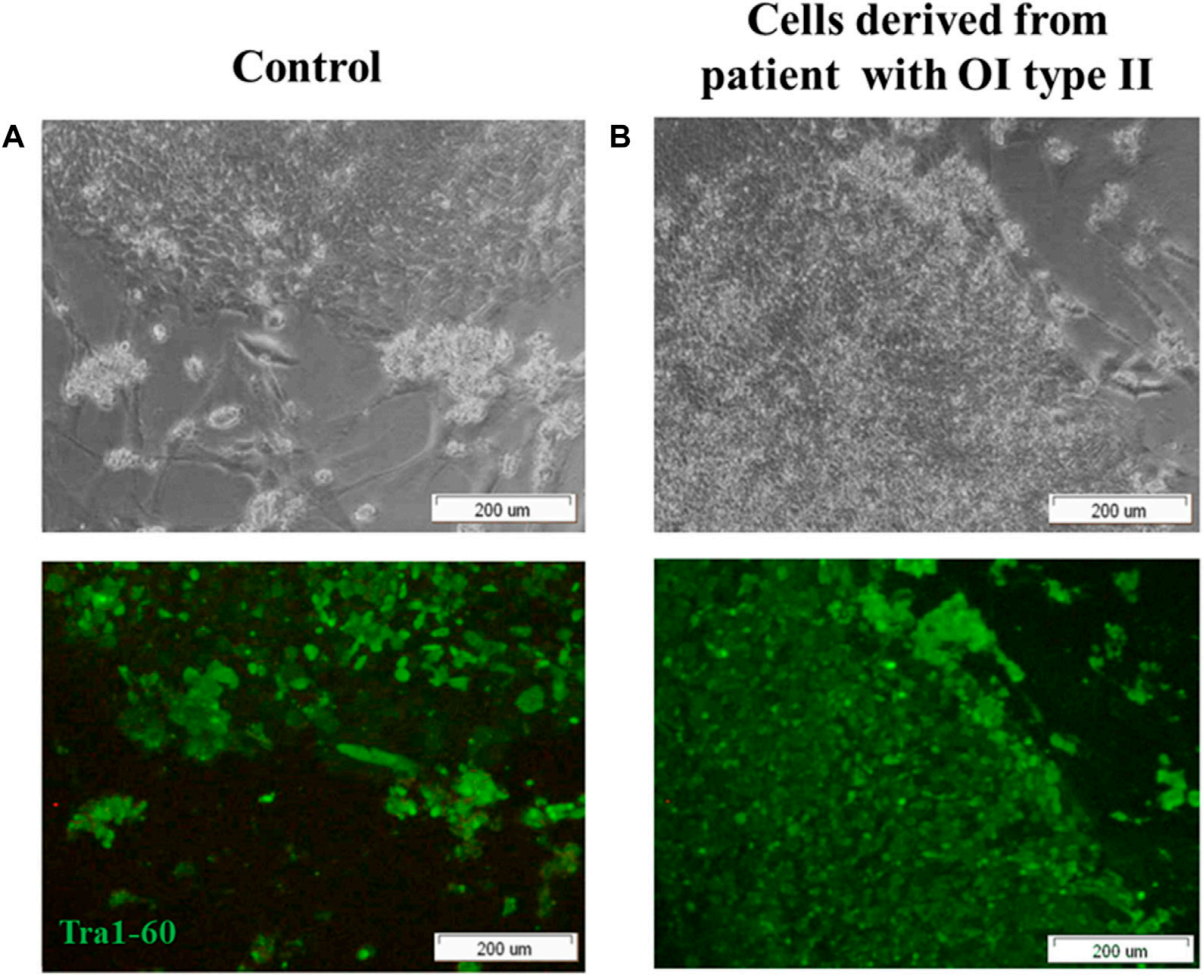
iPSCs obtained from **(A)** control human dermal fibroblasts and **(B)** human dermal fibroblasts with mutation in *COL1A1* gene (del TGGTGCTCC; g:18047–18055 and C>T, (g:18062) reveal pluripotency state. **(A,B)** control human dermal fibroblasts and iPSC with identified mutation show the expression of surface pluripotency marker Tra-1–60 (green fluorescence). The scale bar represents 200 μm. All images are representative of observations from a minimum of 10 colonies. Pictures were taken on day 19 of reprogramming under inverted light microscope (OLYMPUS, Japan).

Fully reprogrammed iPSCs obtained from control and patient cells are valuable tools for further use in gene therapy. To generate iPSCs, Yamanaka factors (Oct3/,4, Sox2, Klf4 and c-Myc; OSKM) were introduced into human dermal fibroblasts using Sendai virus as a carrier of genetic material. Two groups of cells were tested: normal human dermal fibroblasts (control) and human dermal fibroblasts with the two identified point mutations (deletion) in *COL1A1* gene (mutation location: del TGGTGCTCC, g:18047–18055) and substitution (mutation location: g:18062 C>T). Morphological changes were observed in cultured reprogrammed cells within the time of treatment with Yamanaka factors (Supplementary Figure S1). Since day 17, cells started to form round-shaped colonies (Figure 1, Supplementary Materials). We confirmed the pluripotency state in obtained colonies using a specific pluripotency marker: Tra-1–60. Positive immunofluorescence signal was observed in control cells (Figure 1A) as well as in patient-derived iPSCs (Figure 1B).

In order to assess the suitability of the obtained IPSCs for *in vivo* cell therapies, the pluripotent status was investigated, using a set of specific pluripotency markers: SSEA-4 and Tra-1–81. Histograms obtained by flow cytometry analysis represent the percentage of cells that are positive for the antibody.

**FIGURE 2:**
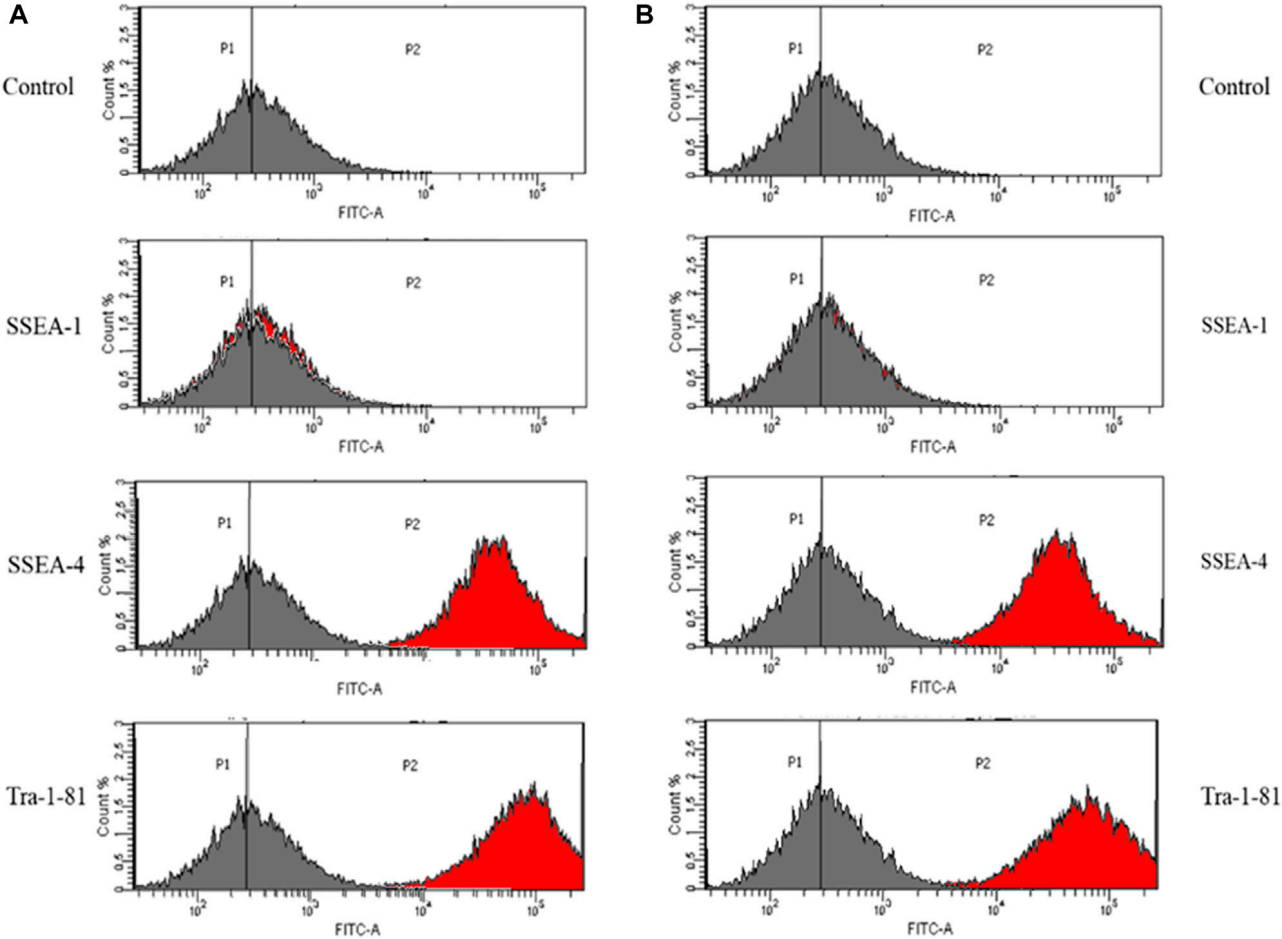
iPSC obtained from **(A)** control human dermal fibroblasts and **(B)** human dermal fibroblasts with mutation in *COL1A1* gene (del TGGTGCTCC; g:18047–18055 and C>T, (g:18062) showed expression of a set of specific pluripotency markers. Unstained control is negative. The grey histogram represents the unstained control and the red histogram shows the number of cells that are positive for the used marker. Vertical lines represent the point where the highest number of cells is indicated.

The analysis revealed that 97,3% of control iPSCs express SSEA-4 and 97,2% are positive for Tra-1–81 (Figure 2A). No differences were detected between control iPSCs and iPSCs derived from patient with a mutation in *COL1A1*. Thus, 97,8% of the cell population was positive for SSEA-4 and 97,3% for Tra-1–81 (Figure 2B).

As a negative control, the detection of SSEA-1 (mouse stage-specific embryonic antigen), which is involved in differentiation but not expressed in undifferentiated iPSCs, was performed. However, SSEA-1 expression was down-regulated after differentiation. With the exception of differentiated iPSCs, adult human monocytes and granulocytes also expressed SSEA-1. Additionally, it has been proven that SSEA-1 play a key role in cell migration and adhesion.

IPSCs obtained from control human dermal fibroblasts and those with the identified mutation were characterized by the expression of pluripotency markers: Stage-Specific Embryonic Antigen-4 (SSEA4) and Tra-1–81. IPSC cells did not show the expression of stage-specific embryonic antigen-1 (SSEA-1) (Bharathan et al., 2017; Inada et al., 2019).

### 3.2 Human skin fibroblasts-derived iPSC with diagnosed OI reveal normal karyotype (46,XX) after reprogramming

Since iPSCs may be generated from any healthy person, iPSCs are considered as a versatile tool for gene therapy as well as regenerative medicine to replace mutated or damaged tissues. It is now well established that remodeling of chromatin structure and an epigenetic changes play a key role in somatic cells reprogramming (Hussein et al., 2013; Baek et al., 2020; Haridhasapavalan et al., 2020). It has been reported that some chromosomal aberrations occur during the reprogramming of somatic cells (Taapken et al., 2011). Although iPSCs show characteristics of germline-type cells, differences in transcription and regulation, both epigenetic and genetic between iPSCs and ESCs have been revealed (Polouliakh, 2013). Chromosomal aberrations detected in iPSCs include insertions, deletions, or changes in the number of copies (Laurent et al., 2011; Liang and Zhang, 2013; Ji et al., 2014). The probable reason for such aberrations is that the reprogramming is accompanied by an increase in reactive oxygen species (ROS) (Kawamura et al., 2009; Ji et al., 2014) which in turn results in the formation of Double Stranded Breaks (DSB) (Liang and Zhang, 2013), posing a threat to genome stability and integrity (Liang and Zhang, 2013). Breaking both DNA strands and non-homologous end-joining cause chromosome fusion and unbalanced translocations (Jeggo and Löbrich, 2015).

**FIGURE 3:**
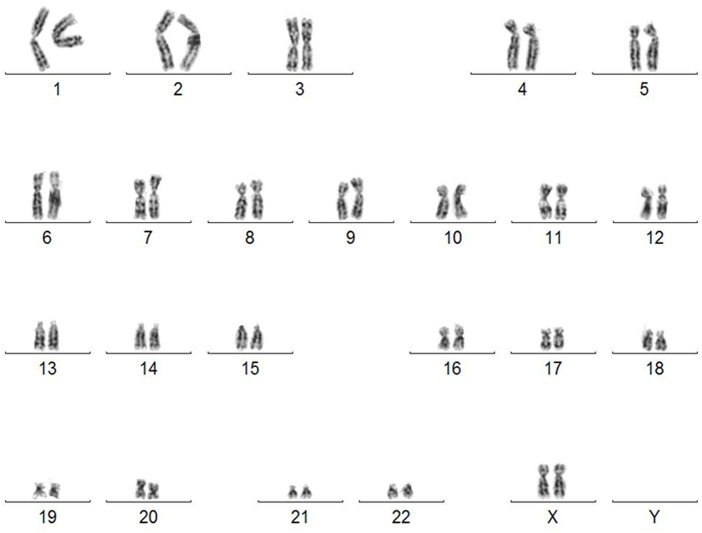
Karyotype of human iPSC derived from skin fibroblasts diagnosed with OI (46, XX). Abnormalities detected in iPSC by G-banding karyotyping have compromised their usefulness in clinical trials.

The lack of genomic aberration is crucial before using such cells in cell/gene therapy. We revealed that the karyotype of the generated iPSC was normal (Figure 3). Karyotype does not reveal any abnormalities even at passage 64 (Supplementary Figure S3). The lack of cytogenetic aberrations and maintaining a stable karyotype of iPSC is a requirement for their potential clinical use.

### 3.3 STAR may be used to deliver DNA into iPSCs and repair mutation in the COL1A1 gene by homologous recombination

Homologous recombination (HR) is essential to access sister chromatids or homologous chromosomes while both strands of the DNA double helix are compromised (Wright et al., 2018). In subsequent experiment we used the STAR polymer to repair DNA mutation in patient-derived iPSC. The polyplex of nucleic acid with cationic STAR polymer was formed by electrostatic interactions between negatively charged phosphate groups of the nucleic acid and positively charged amino groups of the polymer. Star polymer used in this work was obtained using so called “core-first” approach (Mendrek, Fus, Klarzyńska, Sieroń, Smet, Kowalczuk, Dworak), where bromoesterified hyperbranched poly (arylene oxindole) was applied as the macroinitiator of atom transfer radical polymerization of two methacrylates: N,N′-methyldiaminoethyl methacrylate (DMAEMA) and hydroxyl-bearing oligo (ethylene glycol) methacrylate (OEGMA-OH). Its chemical structure is shown in Supplementary Figure S4. The amount of the OEGMA-OH in the star arms, calculated from the proton nuclear magnetic resonance spectrum from the ratio of the DMAEMA:OEGMA-OH, was equal to 10 mol%. The molar mass of STAR, was equal to 100 000 g/mol, monomodal and uniform chromatogram of STAR is shown in Supplementary Figure S5A. The properties of STAR polymer and its behavior in solutions, including media used for biological tests, have been studied in detail in (Mendrek, Fus, Klarzyńska, Sieroń, Smet, Kowalczuk, Dworak) showing that STAR macromolecule does not aggregate significantly in these solutions and its size is small enough (lower than 30 nm) for potential biomedical applications (Mendrek, Fus, Klarzyńska, Sieroń, Smet, Kowalczuk, Dworak). The representative size distribution of STAR in PBS is also shown in Supplementary Figure S5B. STAR polymer showed the ability to form complexes with pDNA, what was determined using agarose gel electrophoresis in (Fus-Kujawa et al., 2020). The complete retardation of pDNA migration was observed for STAR at N/P ratio equal to 6.

Our goal was to create a versatile carrier mix that provides the highest possible transfection efficiency. To evaluate the efficiency of nucleic acid delivery to cells using lipofectamine and STAR polymer, we performed a selection with G418. We expected four variants after transfection: a) cells that do not take neither pDNA nor correct DNA fragment, b) cells that receive only the correct DNA fragment, c) cells that receive only pDNA, d) cells that receive both pDNA as well as the correct DNA fragment. After G418 selection, we expected resistant cells from two variants: with pDNA alone and with both pDNA and the correct DNA fragment. Only these cells survive under G418 selection.

In order to reveal the use of STAR polymer as a versatile nucleic acids delivery tool, we have tested transfection efficiency using STAR polymer alone, lipofectamine only and a mixture of STAR polymer and lipofectamine. We have shown that the use of lipofectamine alone for cells transfection, allows for delivery of nucleic acids with 55% efficiency. Importantly, the use of STAR polymer to transfect the cells resulted in an efficiency of 85%. Most interestingly, we have revealed that the efficiency of nucleic acids delivery using STAR polymer and lipofectamine was 89,5% which was the highest efficiency compared to the other variants (Supplementary Figure S6). Cationic star polymers are an innovative tool for genetic material delivery. Prior to repair mutation, the cytotoxicity of STAR and its polyplex with correct linear DNA fragment and plasmid DNA (pDNA) was also tested. No cytotoxic effect for treated cells was detected (Supplementary Figures S2A and S2B).

**FIGURE 4:**
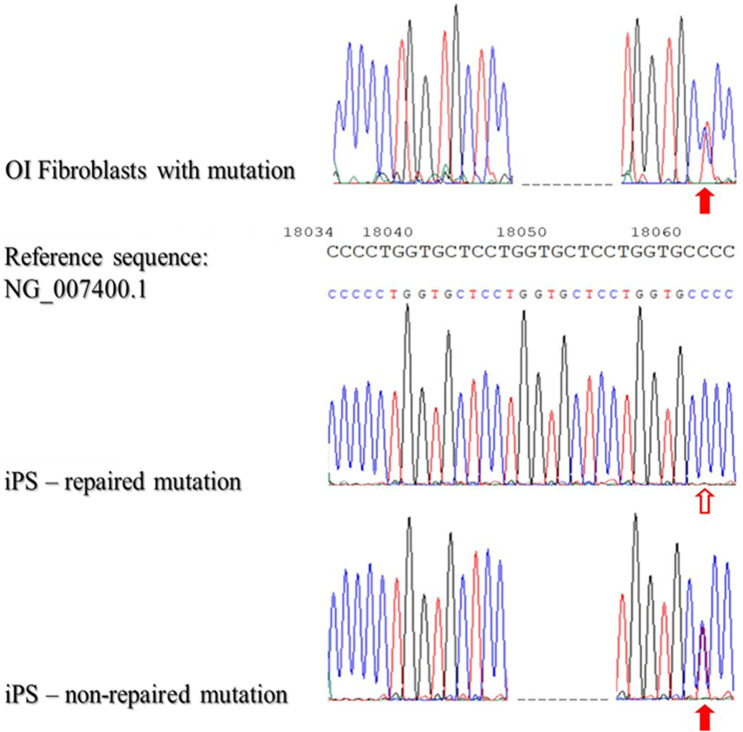
Sequencing of patient-derived DNA repaired based on homologous recombination using STAR polymer as a carrier of genetic material.

Subsequently, the gDNA from patient-derived iPSCs that had not been reprogrammed was then verified to confirm that reprogramming did not result in a repair mutation in DNA sequence. In general, 100 samples of DNA isolated from patient-derived iPSC were analyzed. As the data revealed, DNA mutations were repaired with 84% efficacy using STAR as the nucleic acids carrier (Figure 4).

The presented results of DNA sequencing are representative for sequencing of DNA isolated from fibroblasts with identified mutations (OI Fibroblasts with mutation), repaired iPSCs (iPS-repaired mutation) and non-repaired iPSCs (iPSCs-non-repaired mutation). Red arrows indicate the second mutation - substitution C/T (position - g:18062; non-coding region). The blank arrow indicates the site of the repaired point mutation.

It is known that cationic polymers complex electrostatically negatively charged nucleic acids to form polyplexes, that pass through the cellular membranes. The addition of poly (ethylene glycol) units to the polymeric carrier structure enhanced its aqueous solubility and provided biocompatibility (Gharakhanian and Deming, 2015; Ren et al., 2016). As we have previously reported, the introduction of methacrylates with pendant oligo (ethylene glycol) functions) into polycationic stars, macromolecules significantly reduced the toxicity of polycationic segments, while maintaining high transfection efficacy (Mendrek et al., 2018). In the STAR used in these studies, 10%mol of OEGMA-OH units in its arms ensured no toxicity to HT-1080 cells, which made this macromolecule an innovative carrier candidate for the proposed application (Fus-Kujawa et al., 2020; Mendrek et al., 2018).

### 3.4 *In vitro* differentiation of patient-derived iPSC generates cells with distinct properties

**FIGURE 5:**
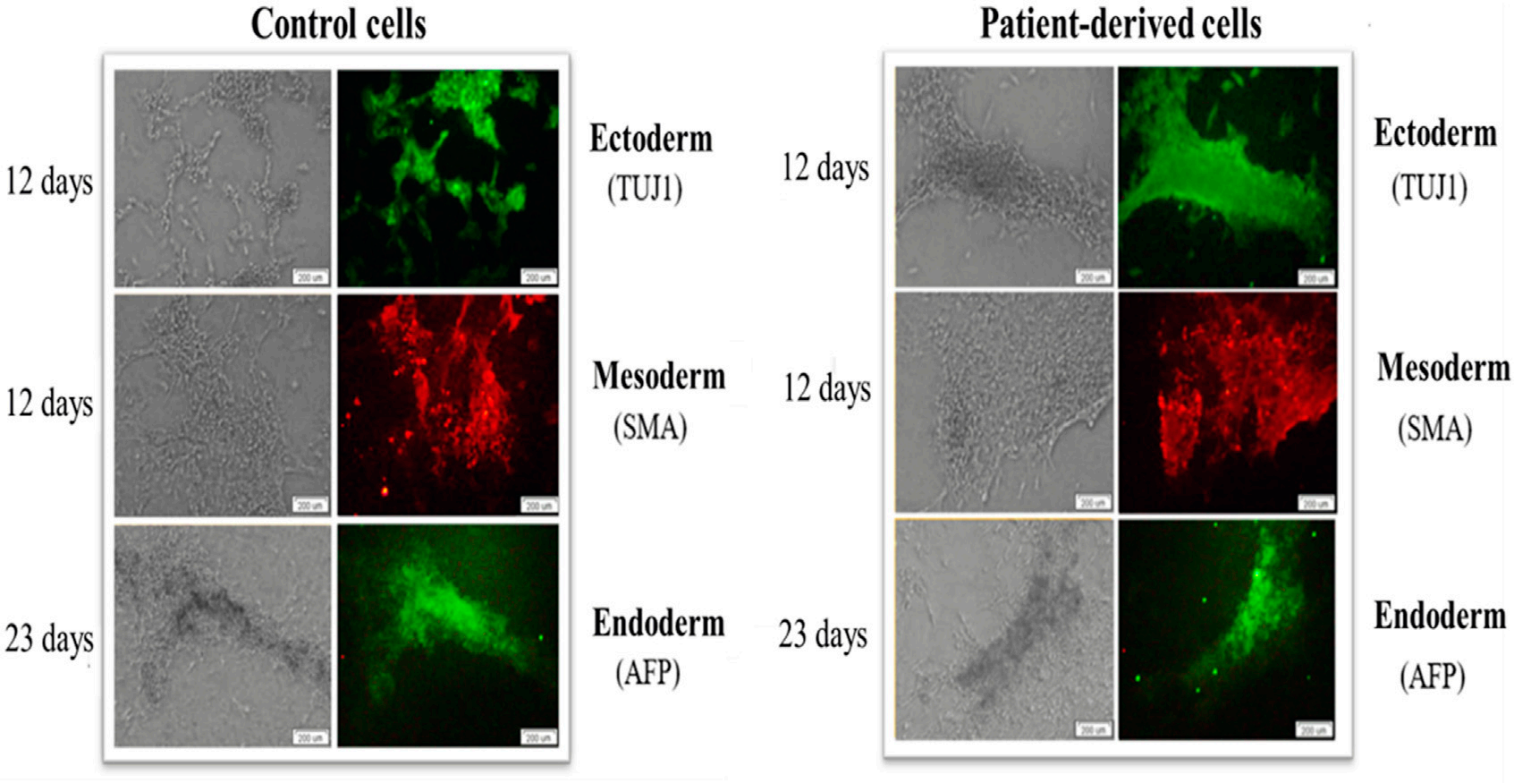
iPSCs show the ability to generate cells of the three germ layers (ecto-, endo- and mesoderm) on days 12, 12 and 23, respectively. Patient-derived iPSCs (right panel) and control human skin fibroblasts (left panel) show the specificity of expression of beta-III tubulin (TUJ1), alpha-fetoprotein (AFP) and Smooth Muscle Actin (SMA). The scale bar represents 200 μm.

This part of the work was based on the hypothesis that the origin of reprogrammed somatic cells is a key factor that may influence differentiation/maturation potential. Importantly, a feature of iPSCs is their ability to self-renewal and differentiation into more specific cell types. IPSCs do not occur naturally and are induced or reprogrammed in somatic cell culture by ectopic co-expression of pluripotency factors. The hypothesis was that the generated iPSCs could form derivatives of all three germ layers. The analyses of experimental data revealed that the generated iPSCs differentiated into cells of three germ layers: ectoderm, mesoderm and endoderm (Figure 5).

**FIGURE 6:**
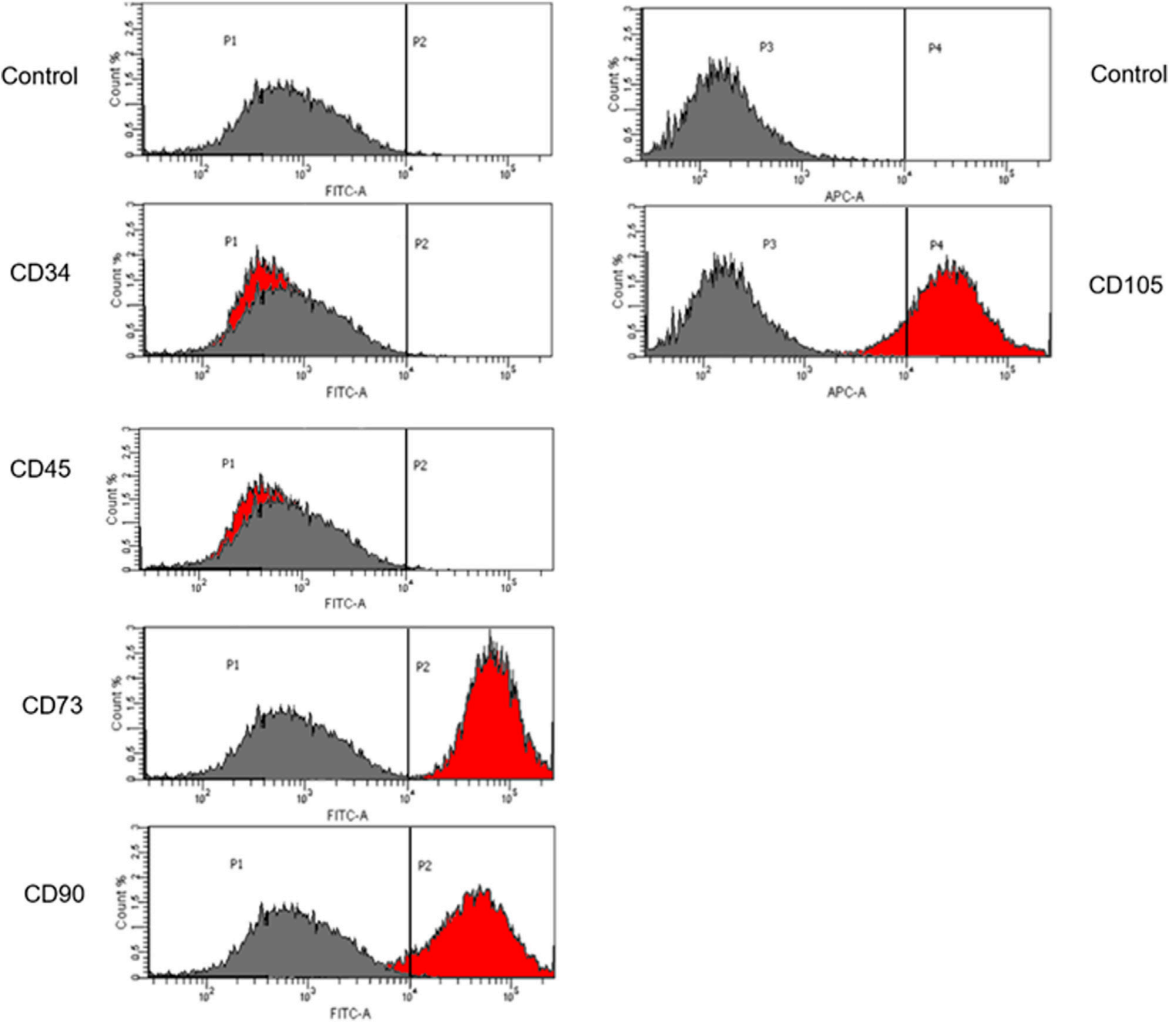
Flow cytometric analysis of iPSCs differentiation. Cells differentiated to MSCs revealed that these cells display the expression of specific Mesenchymal markers: CD73, CD90 and CD105. The unstained control is negative. The grey histogram represents unstained control and the red histogram shows the number of cells that are positive to the analyzed marker.

Corrected iPSCs may be used in gene therapy for patients with defined genetic diseases. The high proliferation rate allows these cells to be used as an infinite source of patient-derived cells. Taking into consideration the fact that iPSCs demonstrate the ability to efficient genetic manipulation, the ability of iPSCs to differentiate into MSCs using differentiation media was assessed. The MSC immunophenotype was confirmed by cytometric analysis with specific markers: CD73, CD90 and CD105. CD34 and CD45 were used as markers for hematopoietic cells (Figure 6). The unstained control was negative.

Appropriate and abundant sources of bone-forming osteoblasts are essential for bone tissue engineering. iPSCs are theoretically an unlimited source of osteoblasts (Zhu et al., 2019). It has been recently reported that osteoblasts derived from differentiated iPSC *in vitro* may be preferred for bone engineering purposes (Duplomb et al., 2007; Lou, 2015; Wu, 2015). iPSCs have been also identified as model for adipocytes (Yao et al., 2020) and chondrocytes generation (Diederichs et al., 2019).

**FIGURE 7:**
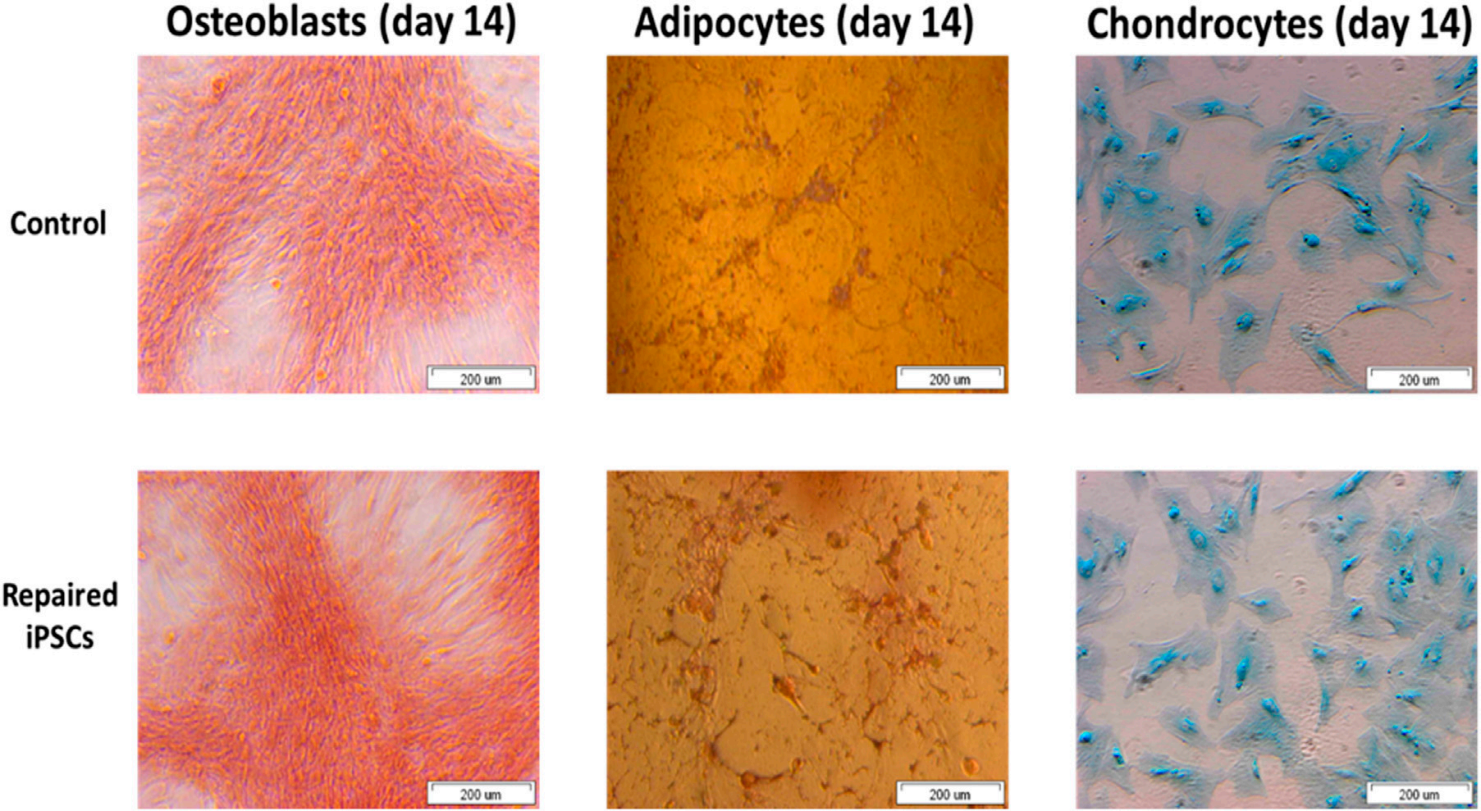
The repaired patient derived iPSC successfully undergo osteogenesis, adipogenesis and chondrogenesis.

We revealed the ability of the iPSCs-derived MSCs for osteogenesis, adipogenesis and chondrogenesis (Figure 7). It allows iPSCs generated from an OI patient to be used as a valuable source of any cell type.

iPSCs reveal the potential to differentiate into osteoblasts, adipocytes and chondrocytes over the time. These cells can be used to generate different cell types for application in gene/cell therapy, drug discovery and disease models. Functionality of iPSCs is a crucial feature of cell-based therapies for or near clinical application.

## 4 Discussion

iPSCs display an unlimited self-renewal capacity and are an abundant source of multiple cell types of therapeutic interest (Yao et al., 2020). The cells are useful for modeling genetic disorders, because of their potential to differentiate into various somatic cell types. They are also susceptible to *in vitro* genetic manipulation, thus enabling pre-clinical assessment of candidate treatment strategies for their performance (Diederichs et al., 2019). However, *in vitro* cultivation and genetic reprogramming increase genetic instability, which can result in chromosomal abnormalities. Maintenance of genetic stability after reprogramming is required for possible experimental and clinical applications. iPSC lines may show clonal and non-clonal chromosomal aberrations in several passages (from P6 to P34), but these aberrations are more common at of cell cultures in later passages (Vaz et al., 2021).

Previous studies have proven the presence of aneuploidies after reprogramming that is most likely associated with errors referred to as mitotic non-disjunction (Ganem et al., 2009). In addition, generated iPSCs may reveal the presence of trisomy chromosomes eighth, 12th, 20q or duplication of 12p. However, the frequency of an extra chromosome 12th, as well as an extra X chromosome was higher in human germ cells, compared to iPSCs (Taapken et al., 2011) Taking it into account, it may be hypothesized that the lack of generated iPSCs may *de facto* prevent to obtain iPSCs with damaged DNA and/or mutations in DNA (Sugiura et al., 2014).

Importantly, the results of the research presented in this work demonstrated that the karyotype obtained from iPSC was normal. This allows us to assume that the induction of pluripotency in somatic cells with Yamanaka factors does not pose a risk of cytogenetic aberrations. This is crucial before considering the use of these cells in *in vivo* therapies (including animal models). However, further studies involving a larger group of patients and other OI types are needed.

The findings presented in the current study are a promising step toward the use of iPSCs in clinical trials. iPSCs can be used as a patient-specific *in vitro* model of genes and other disorders. This creates a unique opportunity to characterize the pathogenesis of a plethora of diseases, such as cancer and other previously incurable diseases. Recently completed clinical trial (Brennan, 2022) has revealed results of studies on the initiation of retinoblastoma (RB) development. *RB-1* deficient iPSCs were differentiated into the retina, to gain insight into the cell of origin for retinoblastoma and cellular events in retinoblastoma tumorigenesis. Another cohort study (Johnson, 2022) has proven the application of iPSCs generated from drawn blood cells and differentiated into cardiac or vascular tissue cells to assess patients’ response to hypertensive medication.

iPSCs models are however mostly considered in genetic diseases. They create means to study both molecular pathogeneses, as well as review potential therapies *in vitro* in cells with the same genetic material as the patients. Currently, iPSC are used as *in vitro* model in numerous clinical trials to explore the pathophysiological mechanisms or therapeutic strategies in human diseases such as Alzheimer’s disease, familial dysautonomia, syndrome, Rett syndrome, genetic cardiomyopathy, or Timothy syndrome. For example, to investigate the molecular mechanisms of genetic cardiomyopathies, the authors generated patient-specific iPSC and then differentiation into neural cells or other specific cells according to the innate error of metabolism (Souidi et al., 2020), while in the case of therapeutic effects, studies largely employed targeted screening to regulate a specific molecular target. Cells obtained in the current study might be used for disease modelling, however the most promising way of iPSCs clinical usage in genetic diseases is patient-specific cell therapy. The underlying concept is to generate iPSCs from the patient’s fibroblast. Then correcting *in vitro* the mutation in DNA sequence causing the genetics disease, as confirmed by these studies, and administering the corrected cells where they are most needed. Therefore, the possibility of treating systemic genetic disorders is particularly attractive.

Various therapeutic and pathophysiological implications of the molecular pathways are known as tools used by stem cells to cope with DNA damage (Vitale et al., 2017). In this research, OI was a model for somatic cell reprogramming and repair using homologous recombination (HR). As HR occurs with low efficacy, the star polymer (STAR) was used to deliver therapeutic sequences to treated cells. An important aspect affecting the recombination efficacy of homologous recombination is also the similarity between the inserted sequence and the DNA of the transfected cells. It has also been shown that a 15-nucleotide similarity between these sequences makes it possible to achieve 90% recombination efficacy when introducing DNA insertion. In contrast, reducing the similarity between these sequences to five nucleotides, reduces the efficacy of the process to 5% (Nagy and Bujarski, 1995; Tarnowski et al., 2010). It can be surmised from this that there is a minimum length of sequence homology that is necessary for efficient recombination. The differences between the sequences act as barrier to homologous recombination (Datta et al., 1997). Most likely, these barriers are related to the removal and/or formation of mismatches in the products of intermediates of recombination (Radman, 1989).

Even though viral vectors are characterized by relatively high efficacy in transducing genetic material into host cells, transfection with these vectors is related to certain serious problems. Their use is associated with modifications that must be performed to derange their replication, assembling, or infection to make them safe. Although, their use may still carry risks of insertional mutagenesis, toxicity and may provoke an immune response. Moreover, there are some limitations in capacity size.

In contrast to the viral vectors, non-viral carriers such as lipids (Gardlík et al., 2005), inorganic particles (Gardlík et al., 2005; Gao, 2012), nanomaterials (Poddar et al., 2022), or polymers (Zu and Gao, 2021) are low in their cytotoxicity, immunogenicity, and mutagenesis. Therefore, they reduce the risk of serious side effects to patients. Additionally, non-viral vectors are more easily manufactured, improving scalability and cost reduction, which is challenging for the production of viral carriers. Though non-viral vectors are much safer than viral vectors, some aspects must be still improved, like gene transfer efficacy or specificity and gene expression duration. The non-viral vectors also provide a larger capacity size and the ability of repeated application. Due to their stability, inorganic materials, especially carbon nanotubes, silica-nanoparticles, and gold and magnetic nanoparticles are one of the non-viral vectors of interest. Nevertheless, their efficacy is low (Gardlík et al., 2005; Gao, 2012; Zu and Gao, 2021). Recently, single-wall carbon nanotubes were shown as gene carriers that significantly improved transfection efficacy. Lipids are biodegradable, and can incorporate hydrophilic and hydrophobic substances. In most lipids, positively charged groups may form electrostatic interactions with genetic material charged negatively and create lipoplexes. However, in the context of lipoplex, low transfection efficacy is challenging (Mendrek et al., 2015; Ren et al., 2016).

Polymeric materials are varied in their features. These materials may be non-biodegradable or biodegradable. Furthermore, the structure of the polymer has an impact on its functionality. The branched polymers are more flexible to molecular modifications on their shape, core-shell microstructure, and multiple end groups of their chains. Mainly polycationic macromolecules are used as polymeric vectors. This feature allows for the concentration of nucleic acids into nano-structures called polyplexes. Simultaneously, the polymer protects the genetic material against degradation and improves the cellular uptake of the polyplex. Moreover, there was found that the polycationic stars showed enhanced transfection in comparison to linear analogues (Zu and Gao, 2021). The number of beneficial features and lack of toxicity speak in favor of the selected star polymer for transfection.

It has been demonstrated that co-opting regulation bypass repair (CRBR) is a gene-correction strategy for monogenic diseases (Hu et al., 2021). This strategy is based on repair in mitotic or post-mitotic cells. CRBR uses non-homologous end junctions (NHEJ) to insert a coding sequence (CDS) and terminators upstream of any mutation in the coding sequence and downstream of the transcription promoter. It comprises a genome editing process that generates a Cas9/sgRNA targeting DSB in the non-coding region of the genome, within the UTR or the intron.

IPSCs may also be used to repair mutation in patients with Huntington’s disease (HD) using the CRISPR-Cas9 homologous recombination system. Generated *HTT* gene knockdown, providing a comprehensive set of isogenic cell lines for testing HD therapeutic drugs. The strategy involves a pair of sgRNAs (HTT_sg1 and HTT_sg4) and the expression of Cas9n from a plasmid as a template. It should be noticed that electroporation efficacy is low and causes cell death and this strategy resulted in no HDR correction (Dabrowska et al., 2020).

Despite CRISPR-Cas9 technology is versatile gene-editing technology, Cas9 targeting specificity is tightly controlled by twenty nucleotide guided sequences of the SgRNA and the presence of PAM (protospacer adjacent motif) next to the target sequence in the gene. Nevertheless, potential off target cleavage activity could still occur (50% chance) on DNA sequence with even three to five base pair mismatches. The effect of off-target can influence the function of a gene and may cause genomic instability. CRISPR-induced Double-Stranded Breaks (DSBs) often trigger apoptosis rather than the intended gene edit. It has been revealed that p53 activation in iPSCs in response to the toxic DSBs introduced by CRISPR often triggers subsequent apoptosis. Then, it may be concluded that successful CRISPR edits are more likely to occur in cells with suppressed p53. Unfortunately, it increases the risk of oncogenic cell survival. It has been also demonstrated that large deletions spanning kilobases and complex rearrangements occur as unintended consequences of on-target activity. It shows a safety issue for clinical applications of CRISPR therapy where DSBs are induced (Ihry et al., 2018; Cullot et al., 2019; Poddar et al., 2020).

In conclusion, our experimental results have demonstrated that somatic cells obtained from patients diagnosed with OI may be reprogrammed by transduction of Yamanaka factors using non-pathogenic Sendai virus as a carrier. Furthermore, we demonstrated the polymers with star-like structures as perfect carriers of therapeutic genetic material. These nanoparticles allow introducing genetic material in order to repair mutations in the *COL1A1* gene by homologous recombination. The use of non-viral carriers to repair DNA mutation provides a novel tool for the safe treatment of inherited and acquired diseases.

Nevertheless, our study has some limitations. More research is needed to evaluate the effectiveness of our approach in gene therapy. It is necessary to perform targeted differentiation of iPS cells into osteoblast cells *in vitro*. Obtaining a stable osteoblast phenotype by iPS cell-derived osteoblasts *in vivo* provides these cells as a viable source for further study in clinical cell therapy for the treatment of OI.

Another challenge will be to grow organs *in vitro* using organoid technology. Compared to typical cell cultures, iPSC-derived organoids better replicate the structural complexity of a real organ, reproducing native tissue architecture, morphology and several biological interactions that occur *in vivo*. The last stage before clinical trials will be *in vivo* tests on an animal model of OI.

There is no doubt that our unique approach to the use of corrected patient-derived iPSCs determines their potential use in *in vivo* therapies with patient-specific cells.

## Data Availability

The datasets presented in this study can be found in online repositories. The names of the repository/repositories and accession number(s) can be found in the article/Supplementary Material.
