# Supplementary figures and images for: Gene-repaired iPS cells as novel approach for patient with osteogenesis imperfecta
Source: Front Bioeng Biotechnol. 2023 Jun 30;11:1205122. doi: 10.3389/fbioe.2023.1205122 (PMC10348904; doi:10.3389/fbioe.2023.1205122)

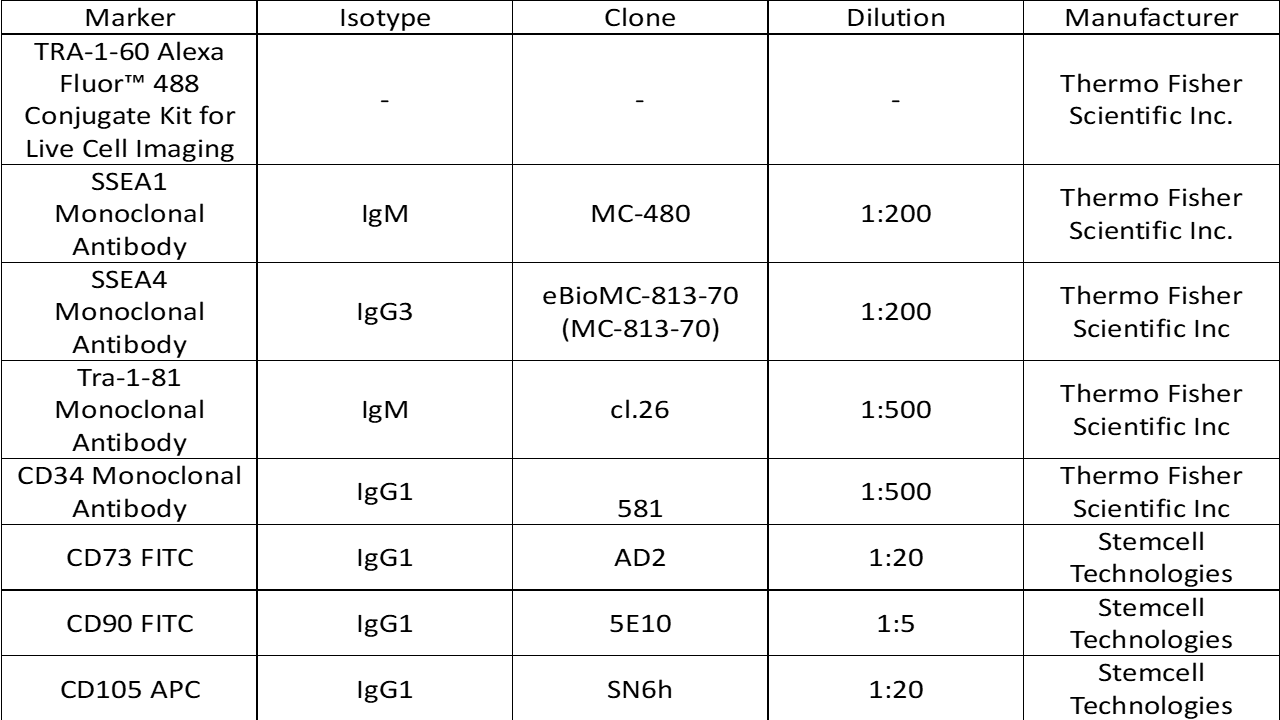

Supplement: Supplementary file 1 [file Image1.TIF]
