# Supplementary material for: Gene-repaired iPS cells as novel approach for patient with osteogenesis imperfecta
Source: Front Bioeng Biotechnol. 2023 Jun 30;11:1205122. doi: 10.3389/fbioe.2023.1205122 (PMC10348904; doi:10.3389/fbioe.2023.1205122)
Supplement: Supplementary file 2 [file DataSheet1.docx]

Supplementary Material

Gene-repaired iPS cells as novel approach for patient with Osteogenesis imperfecta

**Agnieszka Fus-Kujawa*, Barbara Mendrek, Karolina Bajdak-Rusinek, Natalia Diak, Karolina Strzelec, Ewa Gutmajster, Kamil Janelt, Agnieszka Kowalczuk, Anna Trybus, Patrycja Rozwadowska, Wojciech Wojakowski, Katarzyna Gawron**, Aleksander L. Sieroń**

*** Correspondence:** Corresponding Author: [afus@sum.edu.pl](mailto:afus@sum.edu.pl) ; [kgawron@sum.edu.pl](mailto:kgawron@sum.edu.pl)

# Supplementary Figures and Tables

## Supplementary Figures


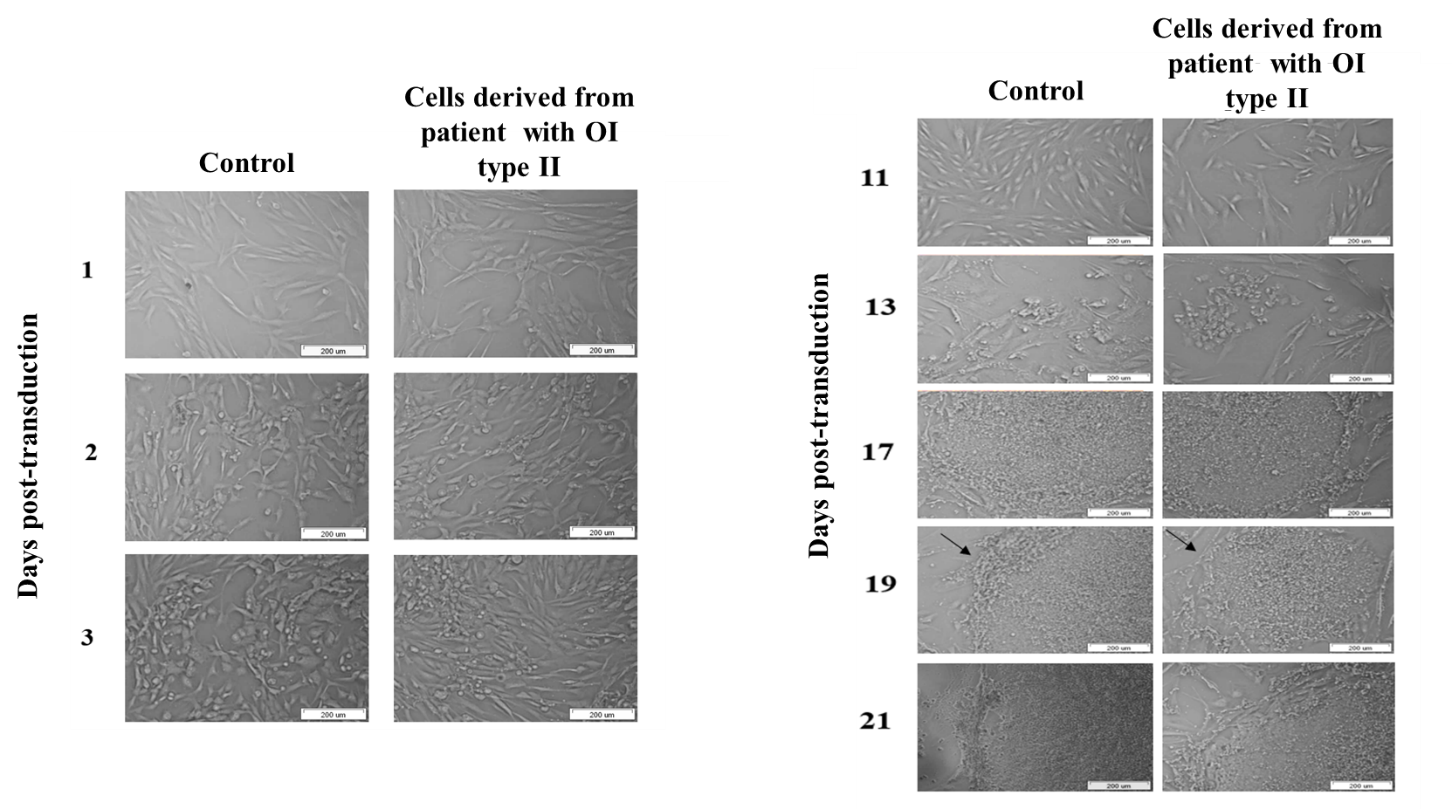


**Supplementary Figure S1. Human dermal fibroblasts derived from patient with *Osteogenesis imperfecta* show morphological changes during reprogramming.**

Pictures of reprogrammed somatic cells demonstrate changes of cells morphology within the time. Small clots has been observed since day 13. Treated cells form colonies at a day 17. The well-formed iPSC colonies are indicated with arrows (day 19). The scale bars in panel represent 200 μm. The images are representative of the analysis of at least 10 views and 3 independent experiments.

**Supplementary Figure S2. STAR and its polyplex do not show the cytotoxicity for induced Pluripotent Stem cells.**

STAR cytotoxicity was assessed before the use in homologous recombination in order to repair mutation in *COL1A1* gene. Polyethylenimine (PEI) was used as a control and shows high cytotoxicity. The cells viability after treatment with STAR polymer reaches 87% for STAR and 82% at the highest N/P for its polyplex with pDNA and correct linear DNA fragment. These results are representative of at least 3 independent experiments.





**Supplementary Figure S3. Karyotype of human skin fibroblasts-derived iPSC with diagnosed OI (46, XX) at passage 64.**

| **Forward primer (5’-3’)** | **Reverse primer (5’-3’)** |
| --- | --- |
| **GCAACACTCCATGACCACAG** | **ACTGCAATCTTCACGGGAGC** |

**Supplementary Table S1. Primers used for amplification of the DNA fragment including mutation site.**


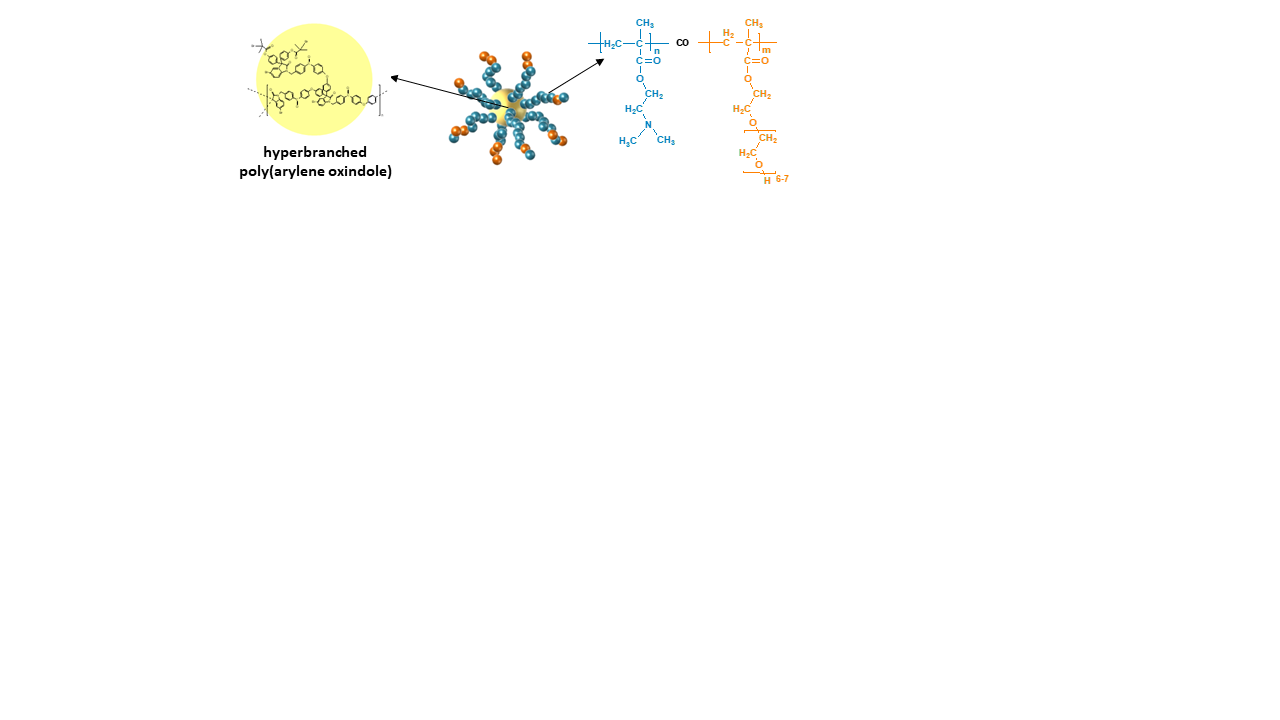


**Supplementary Figure S4. Chemical structure of the star vector.**

**
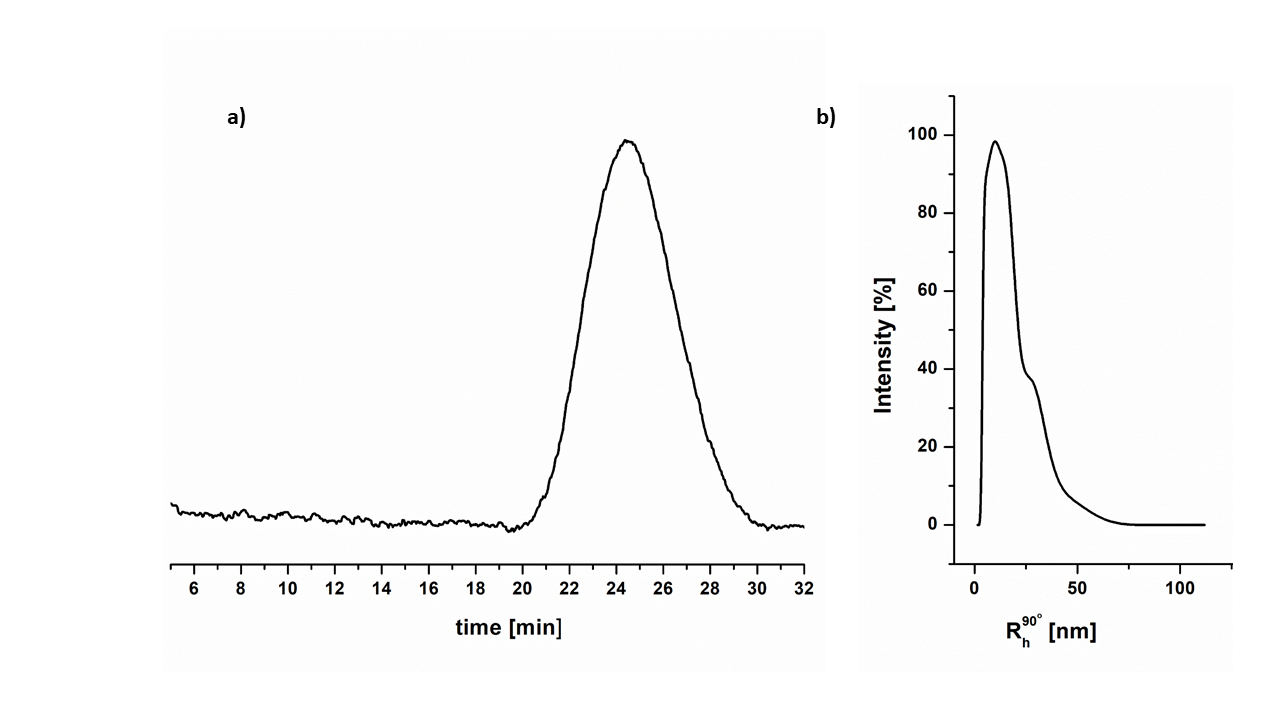
**

**Supplementary Figure S5. Characteristic of the star vector a) chromatogram (RI traces, DMF, 1 mL/min), b) distribution of the hydrodynamic radii (dynamic light scattering, PBS).**

**Supplementary Figure S6. Effects of STAR polymer (variant 1), a mixture of STAR polymer and Lipofectamine (variant 2) and Lipofectamine (variant 3) in a complex with pDNA and the correct DNA fragment on patient-derived iPSCs cells. Transfection was measured as a number of cells that survive under G418 selection and was compared with non-transfected control.**

The datasets presented in this study can be found in online repositories. Please, visit website: <https://ppm.sum.edu.pl/info/researchdata/SUMaa6e933ee3954dbf92f760a3940880e7/>)
